# Alcoholic liver disease: A registry view on comorbidities and disease prediction

**DOI:** 10.1371/journal.pcbi.1008244

**Published:** 2020-09-22

**Authors:** Dhouha Grissa, Ditlev Nytoft Rasmussen, Aleksander Krag, Søren Brunak, Lars Juhl Jensen

**Affiliations:** 1 Novo Nordisk Foundation Center for Protein Research, Faculty of Health and Medical Sciences, University of Copenhagen, 2200, Copenhagen, Denmark; 2 Department of Gastroenterology and Hepatology, Odense University Hospital, Odense C, Denmark; University of Chicago, UNITED STATES

## Abstract

Alcoholic-related liver disease (ALD) is the cause of more than half of all liver-related deaths. Sustained excess drinking causes fatty liver and alcohol-related steatohepatitis, which may progress to alcoholic liver fibrosis (ALF) and eventually to alcohol-related liver cirrhosis (ALC). Unfortunately, it is difficult to identify patients with early-stage ALD, as these are largely asymptomatic. Consequently, the majority of ALD patients are only diagnosed by the time ALD has reached decompensated cirrhosis, a symptomatic phase marked by the development of complications as bleeding and ascites. The main goal of this study is to discover relevant upstream diagnoses helping to understand the development of ALD, and to highlight meaningful downstream diagnoses that represent its progression to liver failure. Here, we use data from the Danish health registries covering the entire population of Denmark during nineteen years (1996–2014), to examine if it is possible to identify patients likely to develop ALF or ALC based on their past medical history. To this end, we explore a knowledge discovery approach by using high-dimensional statistical and machine learning techniques to extract and analyze data from the Danish National Patient Registry. Consistent with the late diagnoses of ALD, we find that ALC is the most common form of ALD in the registry data and that ALC patients have a strong over-representation of diagnoses associated with liver dysfunction. By contrast, we identify a small number of patients diagnosed with ALF who appear to be much less sick than those with ALC. We perform a matched case–control study using the group of patients with ALC as cases and their matched patients with non-ALD as controls. Machine learning models (SVM, RF, LightGBM and NaiveBayes) trained and tested on the set of ALC patients achieve a high performance for data classification (AUC = 0.89). When testing the same trained models on the small set of ALF patients, their performance unsurprisingly drops a lot (AUC = 0.67 for NaiveBayes). The statistical and machine learning results underscore small groups of upstream and downstream comorbidities that accurately detect ALC patients and show promise in prediction of ALF. Some of these groups are conditions either caused by alcohol or caused by malnutrition associated with alcohol-overuse. Others are comorbidities either related to trauma and life-style or to complications to cirrhosis, such as oesophageal varices. Our findings highlight the potential of this approach to uncover knowledge in registry data related to ALD.

## Author summary

Alcoholic liver disease (ALD) is one of the most common chronic liver disease worldwide. It progresses from fatty liver to alcoholic liver fibrosis then to cirrhosis. Unfortunately, people with early-stage ALD have almost no symptoms, for which reason most patients are only discovered when it is already too late. We have thus worked on finding an effective way to detect ALD at an early stage by searching for signs of alcohol over-use among patients. To this end, we analyzed big data from the Danish National Patient Registry, which covers the whole population of Denmark. We found that only 499 patients were diagnosed with alcoholic liver fibrosis and that alcoholic liver cirrhosis is the most frequent form of ALD in registry data. We identified typical diagnoses seen in patients before they developed cirrhosis, many of which relate to the liver not functioning properly. However, we found that patients with fibrosis were much harder to identify based on their past medical records, since most of them show very few signs of being sick.

## Introduction

Alcohol-related liver disease (ALD) is caused by alcohol-overuse [1–3]. It is the third leading preventable cause of death in the world and one of the major chronic liver diseases worldwide [4, 5]. The mortality and morbidity rates from patients with ALD is very high. It accounts for ∼ 500,000 deaths from ALD annually worldwide, representing a huge financial and healthcare burden on society [6]. According to World Health Organization (WHO) Global Information System on Alcoholic and Health (GISAH) [7], the harms related to Alcoholic result in 2016 in some 3 million deaths (5.3% of all deaths) worldwide with more than 2 million deaths in USA. Unfortunately, our knowledge on ALD is still limited by a lack of large population studies [8].

In sufficient quantities, it can potentiate important liver diseases over time such as alcoholic liver fibrosis (ALF) and cirrhosis (ALC). Later, after a long period of liver-cell injury, the fibrosis might progress to cirrhosis and the liver becomes mostly scarred; this progression of the disease takes 10–20 years [9]. Liver fibrosis is a reversible condition, which can regress the harm to the liver caused by alcohol-overused. Cirrhosis, however, is characterized by lasting structural changes to the liver and a reduced ability of the liver to regress fibrosis. Cirrhosis and the accompanying hypertension are the leading cause of liver-related mortality [9]. Complications to cirrhosis include ascites and oesophageal varices, which have their own diagnosis code and are thus available for a registry-based approach like in the present article. Unfortunately, people with fibrosis or reversible early-stage cirrhosis of the liver usually are asymptomatic, that is they do not exhibit any symptoms of disease. Most patients are thus only discovered in late stages when complications to ALC cause the healthcare system to suspect liver disease (75% of patients are diagnosed at a late stage, when 5-year survival may be down to 12% [9]). In this context, we present a machine-learning approach for the early detection of fibrosis or cirrhosis in ALD, that enables to identify at-risk patients with ALF and early cirrhosis before they reach the more advanced stage with complications to ALC [10].

To this end, we use the Danish National Patient Registry (NPR), a country-wide healthcare database that stores patient information such as hospital encounters, discharge dates and diagnoses. NPR is mainly used to handle reimbursement and quality assessment, but is also used in public medical research to assist answering research questions and improve the healthcare process [11–17]. The NPR dataset comprises diagnoses covering the entire population of Denmark during ∼ 19 years (1996–2014). It covers 6.6 million patients and contains ∼101 million unique assignments of primary and secondary diagnoses, which are coded in the International Classification of Diseases 10th Revision (ICD-10) [18], [19]. From this vast and complex NPR data, we provide a knowledge discovery approach that relies on interesting high-dimensional data mining, statistical and machine learning techniques for large-scale, time-dependent data analysis. To identify features predictive of ALD, we perform an in-depth analysis of ALD patients and matched controls by extracting 2-year time windows upstream and downstream of the first ALF or ALC assignment date, i.e. other diagnoses assigned before and after the diagnosis of interest. This is challenging due to the quality issues and many pitfalls of registry data [20].

We present an approach to extract information from NPR helping to discover relevant upstream diagnoses that assist understanding the development of ALF or ALC, and downstream diagnoses that represent its progression into a more complicated situation. Consistent with the late diagnoses of ALD, we show that ALC is the most common form of ALD in the registry data and that ALC patients have a strong over-representation of upstream and downstream diagnoses associated with liver complications, such as ascites and oesophageal varices. We also discover that the small number of ALF patients are less sick than those with ALC. The results of the case–control study performed on the sets of patients with ALC, ALF and their matched non-ALD controls highlight different groups of comorbidities. We find five main groups of comorbid diagnoses that relate to: (i) alcohol-overuse as disorder behavior or diseases of pancreas (ii) under/mal-nutrition as aneamia (iii) liver dysfunction as ascites (iv) trauma and life-style as fracture or type 2 diabetes and (v) upper intestinal or respiratory mucosal diseases as gastric ulcer or hemorrhage from respiratory passages. Some of these comorbidities could be general predictors of ALD.

## Materials and methods

**Fig 1:**
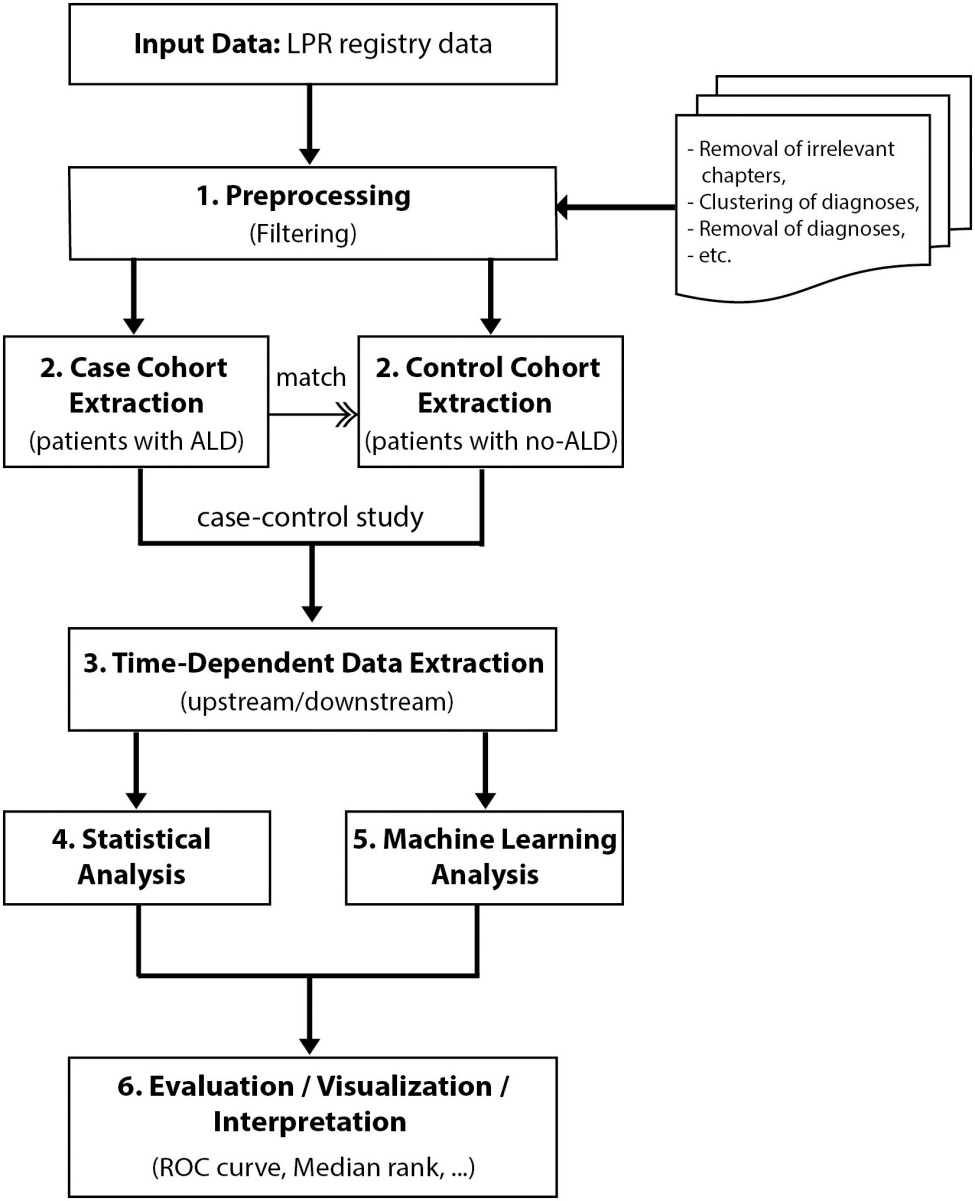
Flowchart of the knowledge discovery approach. The approach is divided into six main steps, which cover data preprocessing, extraction, statistical analysis, and machine learning.

In this section, we present the knowledge discovery approach of Fig 1, built integrating data extraction, statistical and classification techniques. It is divided into six main steps: (i) preprocessing and extraction of the NPR data; (ii) matched case–control study based on the stratification of patients with ALD and on cohorts extracted from random sampling of NPR data in a time-dependent manner; (iii) extraction of upstream and downstream data from cases and controls; (iv) a discrimination analysis based on statistical methods, (v) and a prediction study based on machine-learning techniques; and (vi) evaluation and interpretation of the results.

The whole pipeline was implemented in the Python programming language (version 2.7.12), using mainly scikit-learn (version 0.20.3), pandas (version 0.23.4), and numpy (version 1.15.4) libraries. The experiments were executed on a secure computing cluster.

### Study design and population

The data used in this study is from the NPR, covering all public and private hospital admissions in Denmark in the period 1994–2014. Admissions include all types of encounters: inpatient (admitted to the hospital with overnight stay), outpatient (visit without overnight stay), and emergency-room patients visits. The registry contains administrative information, primary and secondary diagnoses coded according to ICD-10, and operations performed over all hospitals in Denmark [21]. Table A in S1 File defines the list of variables present in the NPR. The dataset includes 6, 631, 923 million patients with a total of 163, 536, 557 million clinical encounters, and contains ∼ 101 million unique assignments of primary and secondary diagnoses. In total, 5, 390, 589 out of the 6, 631, 923 patients were still alive at the end of the study period, 1, 008, 822 died, and 188, 129 patients were inactive (i.e. patients who missed 2 successive annual visits without dropping out) at the end-date of the dataset. In this paper, we use the entire spectrum of diseases from NPR [13, 22].

### Data pre-processing and population

Herein, we perform with the help of medical doctors, a first pre-processing of the NPR data that includes only ICD-10 codes relevant for ALD. This filtering step excludes all referral diagnoses, encounters related to private hospitals as these were only recorded since 2002 [13]. To avoid performing numerous unnecessary statistical tests, with the resulting loss in statistical power, we further filtered out ICD-10 codes from ten chapters (VII, X, XII, XV, XVI, XVII, XVIII, XX, XXI, and part of XIX), which were judged as irrelevant by medical doctors. The full list of these diagnoses is given by Table B in S1 File. Only these specific diagnosis assignments were removed, not the patients per se. We also grouped injury-related codes by type instead of by anatomy. For example, we created one code that combines all fractures (fracture of skull and facial bones (S02), fracture of neck (S12), etc.), since we are more interested in knowing how severely patients were injured than where on the body. In this population-based registry study, we use the ICD-10 level-3 codes (such as R18 for ascites) [13, 23], except for ICD-10 codes directly related to ALD (K70-K77), where we need to be more specific and use level 4 codes (such as K70.3 for ALF) to get the required resolution of all diagnoses related to ALD. The sets of merged codes and level-4 codes are described in Table C in S1 File. The resulting preprocessed NPR data is from hereon referred to as *NPR*_*pp*_.

### Extraction of ALD cases and their matched controls

For the subsequent data analysis, we define three groups of ALD patients: (i) the first group gathers the patients who got assigned ALC only; (ii) the second one contains patients who got diagnosed with ALF but not ALC; (iii) and the third group includes patients who got discovered on time and got diagnosed with ALF before ALC within six months. This time threshold was selected based on the cumulative distribution of the number of days between ALF and subsequent ALC diagnoses (Fig A in S1 File). Such fine-grained stratification of patients is relevant for this study to overcome the bias of population and for Precision Medicine need. The identified groups of patients could then be used as case cohorts for the case–control study (step 2 in Fig 1).

We then need to extract a control cohort from the non-ALD dataset. The selection of population controls is done through an automatic random-sampling process [13] that preserves characteristics required to ensure comparability between the cases and controls [24]. The matching process is performed subject by subject, referring to individual matching, and we select five controls per case to not make negative examples a limiting factor in the subsequent analyses, while also not making the dataset so unbalanced that it would cause problems for machine-learning algorithms. We match the cases with respect to the following characteristics: age (± 10 years), sex, encounter type (describes whether the patient is an in-hospital, out-hospital or emergency-room visit patient), year of discharge (to address any differences in coding over the years) and month of discharge (to correct for seasonal effects) [13]. We use this approach to extract controls from the non-ALD patients for ALF and ALC patients separately.

### Time-dependent data extraction approach

To address the knowledge gap regarding the course of development of ALC for clinical decision making, we need to split the data into upstream and downstream datasets based on the first discharge date of the key diagnosis or outcome (either ALF or ALC). During the data extraction phase, we present a time-dependent data extraction approach that considers different filtering criteria to generate new subsets of data ready for a case–control analysis. Supplementary Table D in S1 File presents the upstream filters applied to extract the case cohorts depending on the outcome of interest (either ALF or ALC). All these filters are applied in order to have a maximal homogeneity between diseased patients of the same mined cohort and to extract a clear and accurate dataset to work on.

The first filter consists in discarding all patients having at least one of the ALD diagnoses in upstream of ALF or ALC to only consider those having as a first ALD diagnosis ALF or ALC. Consequently, we eliminate all patients who were wrongly diagnosed with ALD and then work on a clean set of patients diagnosed with ALF only or ALC only. Since we are primarily interested in upstream diagnoses, inactive or deceased patients are included in the study. The second filter aims to retain only upstream data of ALF and ALC patients, by removing all the downstream data or all the diagnoses assigned the same date or after the first assignment date of the key diagnosis. The third filter is applied to discard all patients for whom we do not have at least 2 years upstream data of the first diagnosis with ALF or ALC, i.e. removal of all patients diagnosed with ALF or ALC less than 2 years after 01/01/1996 (first date of NPR). For the last filtering criteria, we fix 2-year time window of data provided before the first date of the key diagnosis since we aim to find a group of patients not yet having the key diagnosis. We also perform a comparative study of the size of time window to show how it affects the size of the final case cohorts. For the control cohort, we select 2-year data upstream of the first discharge date of the key diagnosis of the matched patient in cases.

Equivalently, to select the 2-year downstream data for cases and controls, we maintain the same set of patients identified during the upstream selection for both cohorts, i.e., for each upstream generated dataset, we extract the list of patients and select their 2-year downstream data from the first discharge date of the key diagnosis. We end up building a feature vector for each patient, where each feature is a vector representing the set of diagnoses the patient got assigned or not. Consequently, we obtain new binary data matrix of the form *Individuals* × *Diagnoses* ready for making data analysis.

### Statistical data analysis

In the following, we perform a statistical data analysis as a first step to examine which diagnoses well separate cases from controls. For this, we rely on: Odds ratio (OR), Statistical significance, Frequency (Freq.), Coverage (COV) and Matthews Correlation coefficient (MCC), to provide different sets of ranked features “*SRF*_*i*_”, where *i* = {1⋯4}.

#### Odds ratio, statistical significance

The OR, as defined in Eq (SE.1), and its associated confidence interval [25] enable determining if there is a relationship between the diagnosis and the target disease. The further the OR is from 1, the stronger is the association between the diagnoses and the disease. Hence, larger values of OR mean that the diagnosis is a good predictor of the development of the disease, but smaller values indicate the opposite. We also need to know if this relationship is statistically significant. Thereby, we calculate the corresponding statistical significance in a two-sided Fisher’s exact test, and we set a significance threshold of *α*. To account for multiple testing, we use Bonferroni correction [26], which controls the probability of making one false positive call. A feature is then judged significant if its corresponding p_value is ≤ *α*/*n*.

#### Coverage

The COV metric is the number of patients among the cases who got assigned a specific diagnosis (defined in Eq (SE.2)). This metric identifies the most frequent features among the patient group of interest, but these features are not necessarily discriminatory.

#### Matthews Correlation Coefficient

The MCC metric [27], defined in Eq (SE.3), is used to measure the correlation coefficient value between two binary variables, in our case having ALF/ALC or not, and getting assigned a given other diagnosis or not. Its values vary between −1 and +1, where +1 is perfect correlation, 0 is no correlation, and −1 is perfect inverse correlation.

#### Data reduction for statistical analysis

Feature selection is used to choose a subset of relevant features from the original feature set. Actually, we are looking to mine statistically significant and relevant features which are highly associated with the outcome. For this, we use as input the new binary dataset of the form *Individuals* × *Diagnoses* on which we perform the sampling approach of the contingency table E in S1 File. Based on this contingency table, we apply the four statistical techniques presented in this section and get different scores for each feature that measure its relevance regarding the disease of interest. We then go through the process of feature ranking by ordering them according to their scores for OR, p_value, COV and MCC. The list is sorted ascending in case of statistical significance and descending for all other metrics. Hence, we obtain four sets of ranked features “*SRF*_*i*_” where *i* = 1⋯4. Finally, we compare the top-ranked features of the four statistical evaluation criteria and select a small candidate subset of relevant and significant features from both statistical and medical points of view.

### Classification for discrimination and prediction analyses

#### Classification models

There are many classification approaches in the literature that can be used to extract relevant features with high discriminatory power. However, choosing the appropriate approach is a challenging step, requiring a careful study and good knowledge of the existing techniques. In the present study, we select popular and widely used supervised classification algorithms for analyzing medical diagnoses in the context of a binary classification problems, as case–control studies. Thereby, we rely on three main classification approaches from which we select popular supervised classification algorithms, appropriate for decision-making problems. These approaches can be categorized broadly into:

1. decision tree classifiers: from the many ensemble decision tree-based learners proposed in the literature [28], we choose two popular, modern algorithms, Random Forest (RF) [29] and Light Gradient Boosting Machine (LightGBM) [30]. RF as an ensemble bagging [31] (also known as bootstrap aggregating) algorithm that applies bagging to decision tree. LightGBM as a high-performance gradient boosting algorithm [32] that applies boosting to decision tree.
2. linear classifiers: besides the large choice of linear classifiers, we select the Naive Bayes (NB) algorithm [33] that assumes a complete independence among features, and the Support Vector Machine (SVM) with its linear kernel algorithm [34].

#### Classification analysis

To identify the set of features discriminating cases from control, that could be predictive of the target disease, we apply four ML algorithms from *sklearn* to the binary data matrix: Random forest (*ensemble.RandomForestClassifier*), LightGBM (*lightgbm.LGBMClassifier*), Naive Bayes (*naive_bayes.BernouilliNB*), and SVM (*svm*). We randomly split the data into a first training set (on average 75% of the samples), and a test set (the remaining 25% of the samples) for estimating the accuracy of the final classification models. We continue by tuning the hyperparameters of each classifier by performing a grid search and optimizing the cross-validated performance on the training data, using GridSearchCV package from sklearn.model_selection. Once the best values have been identified for the hyperparameters, we use the corresponding models as our final set of classifiers. We assess their performance by constructing the Receiver Operating Characteristic (ROC) curve based on the independent test set [35, 36].

#### Prediction analysis on new test set

To test if the predictors already trained on one form of ALD can generalize to others, we use the trained classification models to make prediction on new data selected from the groups of patients with ALD. The selection of this new set depends mainly on which group of patients is used as cases during the classification phase, i.e. if the cases are the patients with ALF, then the new data represents the set of patients with ALC and their matched controls. Otherwise, if the cases represent the group of patients with ALC, then the new dataset includes the group of patients with ALF and their matched controls. Afterwards, we assess the performance of the four fully-trained classifiers on the new selected test set using the ROC curve [35], then we discuss and compare their relative performance during both the learning and the testing phases. This prediction analysis is only performed on the upstream dataset, since downstream diagnoses are obviously not available in the real clinical context when predictions should be made.

#### Analysis of feature importance

To analyse the feature importance, we calculate the relative importance of each feature or diagnosis by using the corresponding importance metric of each applied classifier. Thus, we use the weights of the features for SVM, the coefficients of the features for NaiveBayes and feature importance for both tree-based classifiers. The scores given by these metrics enable the ranking of the features and the assessment of their relevance after classification. Then, we sort the features by relevance to obtain an optimal subset of top-scoring predictive ones. In total, four subsets of ranked features are generated and combined to compute their median rank [37]. One more time, we sort the combined features w.r.t their median rank values and present the top ranked ones to medical doctors to underline if possible the general ALD predictors [38].

## Results & discussion

### Groups of patients with ALD

From the preprocessed registry data (*NPR*_*pp*_), we derive two main subcohorts: (i) a first subcohort of 33, 391 ALD patients; and (ii) a second subcohort of the remaining 6, 010, 942 non-ALD patients with 63, 416, 907 clinical encounters. By looking at the individual diagnoses of the patients in the subcohort of patients with ALD, we get six main ICD-10 codes. Each code is part of the the block “Diseases of liver” (K70-K77) and represents a type/stage of ALD. ALC is the most common form of ALD in the NPR data, with 23, 271 patients who got diagnosed with ALC out of the total of 33, 391 patients with ALD. By contrast, we find only 499 patients with ALF (also called alcoholic fibrosis and sclerosis of liver) among the ALD patients. For the remaining ICD-10 codes of ALD, we get 5, 959 patients with Alcoholic fatty liver, 4, 275 patients with Alcoholic hepatitis, 5, 546 patients with Alcoholic hepatic failure and 5, 823 patients with Alcoholic liver disease-unspecified (the same patient can be counted under multiple codes).

### Three main groups of patients with ALF and ALC

**Fig 2:**
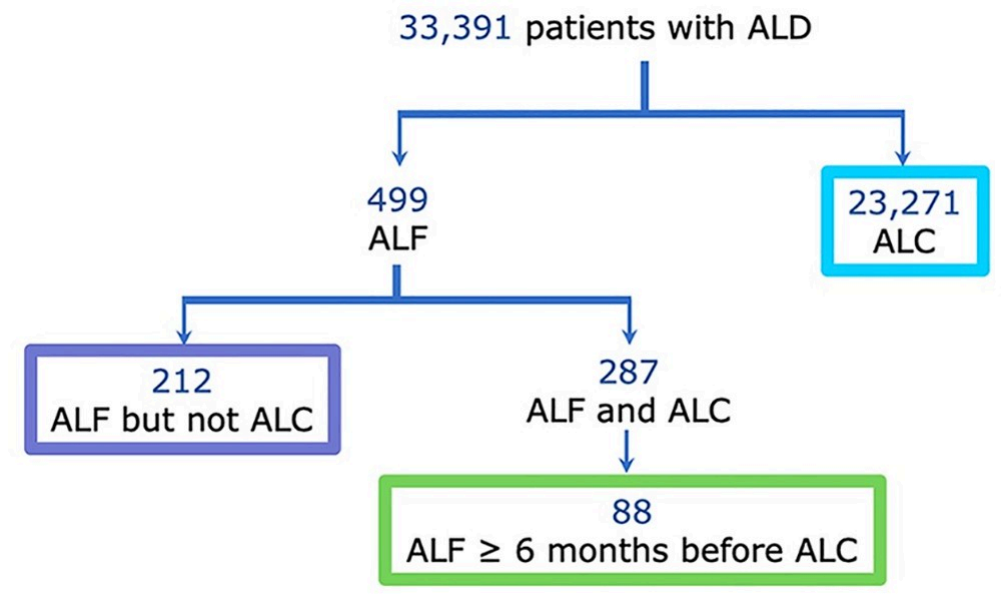
The number of patients with ALF and ALC obtained from the filtering process. Three main groups of patients are identified representing patients with ALC, patients with ALF but not ALC, and patients diagnosed with ALF at least six months before being diagnosed with ALC.

We stratify the 499 patients with ALF and obtain: a first group of patients who got diagnosed with ALF but not with ALC, a second group of patients who got assigned both ALF and ALC codes, from which we extract the third subgroup of patients who got assigned the ALF code less than six months before ALC. Consequently, the classification of patients with ALD led to the identification of three main groups of patients: (i) 23, 271 patients with ALC only, (ii) 212 patients with ALF but not ALC, and (iii) 88 patients who got diagnosed with ALF at least 6 months before ALC (Fig 2). To get a clean sets of patients and extract a 2-year upstream and downstream time window of the first assignment date of ALC or ALF diagnosis, we apply the filters of Table D in S1 File. New smaller sets of patients are derived: (i) 9, 373 of patients with ALC; (ii) 12 patients with ALF but not ALC; and (iii) 23 patients with ALF at least 6 months before ALC. A flowchart describing the number of patients with ALC at each filtering step is given in Fig B in S1 File. It shows the first filter as the main discarding criteria since the number of ALC patients is largely reduced at this step (from 23, 271 to 14, 746 patients with ALC).

Additionally, we explain in Table F in S1 File how the size of the time window influences the final results when processing the 2nd and 3rd filters of Table D in S1 File. It highlights that more patients are considered by using a time window of 2 years (8, 256 patients for 6-year upstream data vs 9, 373 patients for 2-year data), and lower number of ICD10 codes are identified for the same dataset (871 of ICD10 codes for 6-year upstream data vs 811 codes for 2-year data). The increasing of the number of patients when reducing the size of the time window is explained by the application of the 3rd filter. By selecting a time window of 6 years, a lot of patients diagnosed with ALC less than 6 years after 01/01/1996 are removed, but more ICD10 codes are extracted. Also, by selecting a time window of 1 year, we obtain smaller number of patients (8, 626) and codes (750) than when selecting 2 years of upstream data. This decreasing number of patients is explained by the fact that many patients did not have any contact with the hospital within the year preceding the first diagnosis with ALC. The final numbers of ALF and ALC patients underline the main motivation for this work, namely that the vast majority of ALD patients are discovered only after ALF has progressed into the irreversible ALC.

To our surprise, only 12 ALF patients did not progress to ALC, for which reason it is not statistically possible to derive relevant conclusions about the most discriminant diagnoses among this group of interest. We then compute the most frequently assigned diagnoses among these three groups of patients within a 2-year time windows upstream of their first ALF or ALC diagnosis. We find that the group of patients who were diagnosed with ALF and never developed ALC appears to be much less sick than the two groups of patients with ALC. ALF patients are mainly over-represented by life-style diagnoses such as type 2 diabetes and hypertension. Both groups of ALC patients reveal a strong over-represented by diagnoses caused by liver diseases, such as ascites and oesophageal varices. Table G in S1 File gives an overview of the list of most frequent features among ALC patients. Fig C and D in S1 File show the histograms of top-10 most frequent upstream diagnoses among ALC and non-ALD patients, respectively. They both share common diagnoses as fracture, open wound, superficial injuries and type 2 diabetes mellitus.

Based on the group of patients with ALC (9, 373 patients), we select their matched controls (5 controls per case) from the set of patients with non-ALD (6, 010, 942 patients) for a case–control study. We then extract 2-year upstream data from controls and consider as starting timing point, the first date of ALC assignment of their respective matched cases. This resulted in 9, 082 ALC patients that match 43, 844 controls. For downstream data, we use the same set of patients and further require 2-year downstream data, resulting in 6, 311 ALC patients and 30, 696 controls. The number of clinical encounters for upstream and downstream varies between 68, 029 and 96, 728 (see Table H in S1 File), considering only those involving diagnoses that remain after the filtering steps. ALC patients have an average of 4.38 unique diagnoses within a 2-year upstream window, whereas the controls have an average of 1.82. It is not surprising that the controls have fewer unique diagnoses than ALC patients, since diagnoses judged as irrelevant to ALD were filtered out before counting. The age and sex distribution of all the groups of patients are shown in Fig E and F in S1 File, respectively. The majority of the ALC patients, and their matched non-ALD controls, are male (67%) and between 45 and 80 years old. By contrast, the 12 patients with ALF stand are mainly women and younger than the ALC patients.

To identify the small set of features that have an impact on the development and/or progression of ALC, we select both 2-year upstream datasets of the set of patients with ALC and their matched controls. We then build a new data representation space made of binary tables whose dimensions for cases (ALC) and controls (non-ALD) are respectively equal to 9, 082 patients × 820 codes and 43, 844 patients × 941 codes. Their combination gives a big unique binary upstream dataset of 52, 926 patients and 969 unique codes. We use this case–control dataset as a basis to make the statistical and discrimination/prediction analysis.

### Six upstream groups of comorbidities according to statistical analysis

Before making the statistical analysis, we firstly discard rare diagnoses assigned to less than 8 patients, and obtain 652 codes out of the 969. Among these remaining codes, we identify 256 significant ones with a *p*_*value* < 0.001. We compute their OR, its confidence interval, COV and MCC and obtain different sets of ranked codes depending on the metric of interest. However, the features with high OR are not necessarily the most frequent ones with the highest coverage values. Thus, we select the 20 top-ranked features according to MCC, OR, and COV that we list in Table G in S1 File and rank according to MCC. However, to extract the important features, we rely on the top-20 best features according to MCC, since it is a good compromise between both computed metrics OR and COV.

The top-20 diagnoses upstream of ALC fall into six groups: (i) diagnoses with the strongest associations to liver dysfunction; (ii) diagnoses, other than liver diseases, which are often caused by alcohol and minor psychiatric conditions; (iii) diagnoses related to under-nutrition, that are neither caused by alcohol nor by liver disease; (iv) diseases related to trauma and injuries; and (v) conditions in the upper gastrointestinal tract and finally (vi) other diagnoses. Table 1 presents the six categories, and statistically evaluates the upstream diagnoses of each one. All the selected diagnoses are statistically significant with a corrected *p*_*value* < 0.001 and an *OR* > 2. Their MCC values vary between 0.02 and 0.44, showing a positive correlation with the development of ALC among the patients.

**Table 1:** Groups of statistically significant (*p*_*value* < 0.001) upstream diagnoses ranked according to their MCC values. For each diagnosis, we show its values for MCC (Matthews Correlation Coefficient), OR (Odds Ratio), OR_CI (its confidence interval) and COV (Coverage).

ICD10 code	MCC	OR	99.9% OR_CI	COV
**1**. **Diagnoses related to liver dysfunction**				
Fibrosis and cirrhosis of liver	0.28	140.99	80.51-246.87	943
Ascites	0.28	67.63	45.83-99.81	977
Other diseases of digestive system	0.26	26.03	20.21-33.54	1,007
Oesophageal varices	0.25	141.21	74.00-269.44	727
Other diseases of liver	0.17	40.71	24.75-67.46	388
Hepatic failure, not elsewhere classified	0.16	67.97	33.85-136.48	326
Chronic viral hepatitis	0.13	20.24	12.86-31.86	269
**2**. **Diagnoses related to alcohol-overuse**				
Mental-behavioral disorders due to alcohol	0.44	20.25	17.87-22.94	3,075
Other diseases of pancreas	0.13	10.30	7.60-13.96	364
Other and unspecified polyneuropathies	0.12	19.36	12.19-30.75	250
Symptoms and signs involving appearance and behaviour	0.02	19.32	1.43-260.6	8
**3**. **Diagnoses related to malnutrition**				
Other anaemias	0.18	8.63	7.07-10.54	748
**4**. **Diagnoses related to trauma and injuries**				
Fracture	0.13	2.68	2.41-2.98	1,709
Superficial injuries	0.10	2.28	2.02-2.57	1,215
Open wound	0.09	2.19	1.94-2.46	1,269
**5**. **Diagnoses related to upper intestinal mucosal**				
Gastritis and duodenitis	0.17	11.01	8.57-14.14	560
Gastric ulcer	0.14	9.68	7.31-12.82	410
Duodenal ulcer	0.12	9.64	6.98-13.32	310
Oesophagitis	0.11	10.96	7.44-16.13	235
**6**. **Others**				
abdominal and pelvic pain	0.10	3.56	2.96-4.27	578

The first group includes six diagnoses with the highest OR, which are either complications to liver cirrhosis or codes that represent obvious signs of severe liver disease. While they appear as upstream diagnoses in our analyses in the sense of which was discovered and coded first, medically they are downstream consequences of ALC. This is either caused by coding complications to ALC prior to the proper diagnosis of ALC. Or firstly assigning liver failure diagnoses to patients before knowing their alcohol overuse [39].

The second group consists of diseases that, like ALC, are caused by alcohol. Despite the obvious example of mental diseases due to alcohol which is the most frequent diagnosis [40], the most interesting example is other diseases of the pancreas, which we believe reflects a vague coding practice for harm to the pancreas caused by alcohol overuse. The disease ‘alcoholic polyneuropathy’, which is also caused by alcohol, is the most frequent among those affected to the diagnosis ‘other and unspecified polyneuropathies’. The diagnosis related to symptoms and signs involving appearance and behavior is used for assigning a code to a patient’s condition in the cases where medical examination did not find any disease and when the doctor believes that the cause of the health care contact is best described caused by one of the conditions coded in this category. A notable and frequent member of this category is the code bad personal hygiene. Although it could be due to other causes than alcohol, we believe that this coding is reflective of the social decline that is sometimes related to heavy alcohol overuse.

The third group includes associations related to malnutrition or under-nutrition [41]. The code ‘other anaemias’ that appears in upstream, reflects anemia due to malnutrition caused by lack of vitamins and minerals such as vitamin-B12. We notice that alcohol-overusing patients substitute regular food with alcohol and that this results in a poor nutrient intake that explains this finding [42], [43].

The fourth group contains diseases related to trauma and injuries. Drunkenness is a known risk-factor for enduring trauma, which sometimes lead to fractures, superficial wounds or open wounds.

The fifth group contains diagnoses for damage of the mucosal membranes of the upper intestinal tract. Alcohol is a known risk factor for these conditions. Diagnoses representing ulcers of the gastrointestinal tract reflect that patients with ALC has a greater tendency to have a history of upper gastrointestinal bleedings. At a later state in their course of disease when they have progressed to cirrhosis and the complication of varices of the oesophagus, they might as well get upper gastrointestinal bleeding from the oesophageal varices. As shown later in the downstream diagnoses, however, they maintain the tendency to bleed from ulcers which are not caused by the liver disease but by the alcohol overuse that also causes the liver disease [44].

The last group includes only the coding of abdominal and pelvic pain. This code might be assigned to subclinical cases of alcoholic pancreatitis, i.e. patients with abdominal and pelvic pain may have an undetected alcoholic pancreatitis during their examination [45].

### Six upstream groups of comorbidities according to ML analysis

#### The four classification models perform similarly

**Fig 3:**
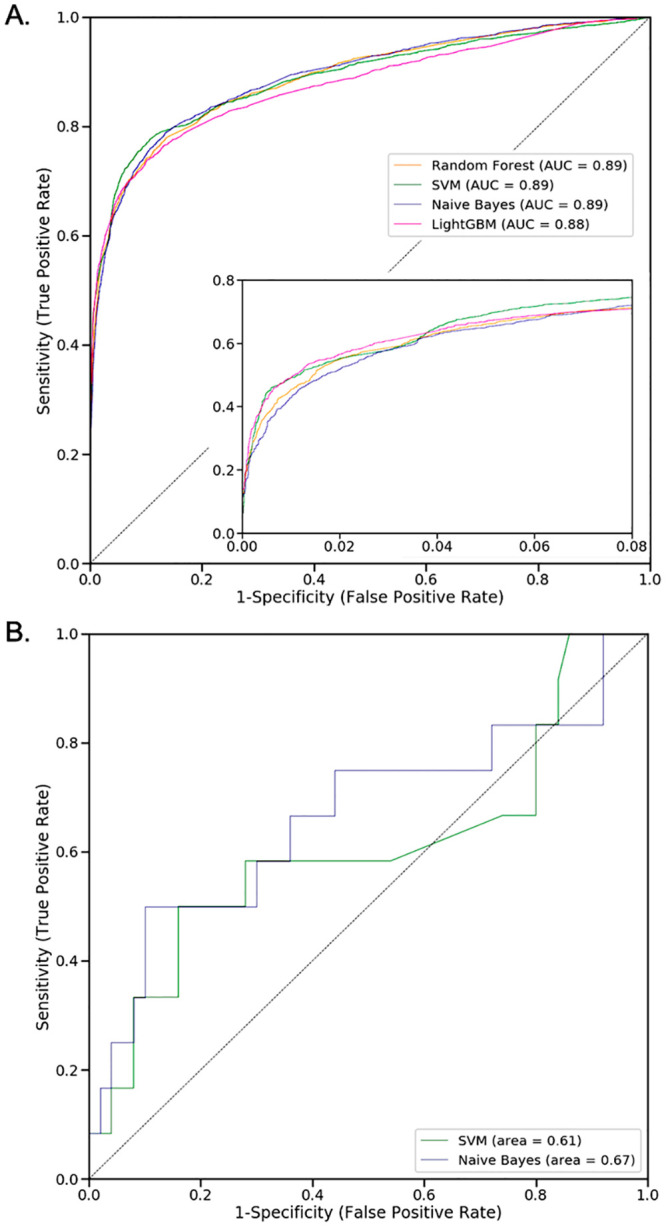
The performance of the classification models according to the ROC curves. [**A**.] presents the independent test-set performance for ALC patients and their matched controls; the insert shows a zoom of the low-false-positive part of the curves. [**B**.] presents the ROC curves of the SVM and NB models trained on the set of ALC patients, and tested on the ALF patients and their matched controls. The RF and LightGBM models were found to not be applicable to ALF patients (see main text). In both panels, the dashed line shows the random performance of a model with no discriminatory power.

To perform a machine learning analysis, we use as input the whole upstream binary dataset and we apply the four selected ML algorithms: Random forest, LightGBM, SVM, and Naive Bayes. The entire binary dataset of ALC and non-ALD patients is partitioned into a training set (*39,694 patients* × *969 codes*) for training the classifiers, and an independent test set (*13,232 patients* × *969 codes*) for assessing the classification performance (Fig 3A). It compares the four classifiers in the ROC plot by showing in a graphical manner the trade-off between clinical sensitivity and specificity for all potential cut-offs for a test. We show both the full ROC curves and a zoom on the low false-positive-rate part, which is most relevant for clinical applications. Reassuringly, the AUC scores show that all of the ML algorithms perform about equally well on the same data set. They all have a AUC = 0.89, except LightGBM classifier that has a very close AUC score equal to 0.88.

#### NaiveBayes, the best performing classifier on the test set of ALF patients

To underline if it is possible to identify general ALD predictors, we consider the four classification models trained on the set of ALC patients and test them on the small set of 12 ALF patients. For that, we select the set of 12 ALF patients and their 5 matched controls and we transform the representative space into binary. We obtain a second binary dataset representing ALF patients of the form *60 patients* × *969 codes*. Only 60 patients got discovered due to the lack of matching 5 controls per case. Then, we test the four trained classifiers on the generated ALF binary dataset and evaluate their performance based on the ROC curve of Fig 3B. The latter shows that the performance of all the classifiers obviously drops a lot compared to Fig 3A. The Naive Bayes is the classifier that drops the least with an AUC equal to 0.67, followed by SVM which has an AUC of 0.61. Both tree-based classifiers (RF and LightGBM) are unable to score the majority of the ALF patients, as they selected features (ICD-10 codes) that are not observed in ALF patients and their matched controls. Conversely, the Naive Bayes and SVM methods assign weights to all input features, for which reason they can be applied to the ALF dataset. Accordingly, Naive Bayes is the best classifier that generalizes to ALF.

#### Quite similar groups of upstream comorbidities compared to the statistical results

To gain insights into how the classifiers work, we compute the feature importance with regard to each classifier and compare the feature lists to the results of the statistical results. For SVM, we use the feature_weight’ scoring metric. For RF and LightGBM, we consider the Gain-based metric. For Naive Bayes, we evaluate the features according to their coefficients. We sort the features from each model according to their relevance scores and compute their median ranks across all the models. Table 2 lists the top-20 features from machine learning according to median rank. All these discovered features are statistically significant with p_value < 0.001. The given table also ranks the remaining features w.r.t. each model and classifies them into six main groups of comorbidities.

**Table 2:** Groups of top-scored upstream diagnoses. They are identified from computing the Median rank on the basis of the results of RF, SVM, LightGBM (LGBM) and NaiveBayes (NB), trained and tested on the set of patients with ALC and their matched controls.

ICD10 code	Median	RF	SVM	LGBM	NB
**1**. **Diagnoses related to liver dysfunction**					
Ascites	2	2	2	2	6
Other diseases of digestive system	4	3	9	3	5
Fibrosis and cirrhosis of liver	4	4	1	4	7
Oesophageal varices	5	5	3	5	11
Other diseases of liver	8	8	5	8	18
Hepatic failure, not elsewhere classified	9	10	4	9	22
**2**. **Diagnoses related to alcohol-overuse**					
Mental-behavioural disorders due to alcohol	1	1	11	1	1
Other diseases of pancreas	17	11	57	16	19
Other and unspecified polyneuropathies	22	12	15	757	29
Syncope and collapse	25	26	180	19	25
**3**. **Diagnosis related to malnutrition**					
Other anaemias	8	6	69	7	10
**4**. **Diagnoses related to injuries & life-style**					
Fracture	6	7	231	6	2
Superficial injuries	13	17	294	10	4
Open wound	13	14	244	11	3
Type 2 diabetes mellitus	21	24	400	18	9
**5**. **Diagnoses related to upper intestinal or respiratory mucosal**					
Gastritis and duodenitis	12	9	174	12	13
Gastric ulcer	15	13	158	15	15
Duodenal ulcer	19	16	168	14	23
Hemorrhage from respiratory passages	21	18	74	13	24
**6**. **Others**					
Abdominal and pelvic pain	18	19	225	17	12

The first group is related to liver damage. It includes diagnoses already identified by the same group from statistical results. The second group contains a new diagnosis related to alcohol over-use, which is ‘syncope and collapse’. This diagnosis is used when someone has been admitted to hospital because either he has fainted or because doctors did not find a medical cause for the loss of consciousness. This may have several reasons, but in our case, we believe that this loss of consciousness is highly associated with drunkenness or other substance use, when no other explanation is given. The third group contains only one diagnosis which was already discovered by the statistical analysis, namely ‘other anaemias’ caused by malnutrition. The fourth group includes both conditions caused by trauma (as fracture, superficial injuries and open wound), and lifestyle diseases (as type 2 diabetes). We group the latter diagnosis together with accidents because the incidence of type 2 diabetes is highly affected by people’s lifestyle. According to Table 2, these diagnoses are highly ranked by the NaiveBayes, the classification model that best generalizes to ALF. They may then have the strongest predictive power of ALD development with comparison to the others groups. The fifth group related to upper intestinal or respiratory mucosal contains one additional diagnosis, which is ‘hemorrhage from respiratory passages’. The connection between these bleedings and alcohol is less obvious from a medical perspective. The last group shows the same diagnosis as for the statistical results related to ‘abdominal and pelvic pain’.

Importantly, by using machine learning classifiers to analyse 2-year upstream data of patients with ALC and others with ALF, we discover a subset of features capable of detecting ALC patients and that could be promising in predicting ALF.

### Downstream comorbidities

To identify the list of discriminant diagnoses that represent the progression of ALC to liver failure, we perform a case–control study and work on 2-year downstream time window of the first assignment date of ALC for cases and their matched controls. For this, we consider the set of 6, 311 patients with ALC as cases and the set of 30, 696 patients as controls. Then, we construct a new data representation space made of their binary tables that we combine to get a new downstream unique binary matrix of 37, 007 patients and 959 unique codes. This new matrix is then used as input to make only the statistical analysis since we are not looking for predictive features in downstream of first ALC. For this, we proceed as for the upstream statistical analysis, we compute the p_value, the OR and its confidence interval, the Coverage and the MCC based on the downstream binary dataset. We then select statistically significant features with a corrected p_value lower than 0.001, which are on the top-20 according to MCC. These features are presented in Table 3. The top-10 most frequent downstream diagnoses among ALC and non-ALD patients are given in Fig G and H in S1 File, respectively. Both lists contain the diagnoses fracture, type 2 diabetes mellitus, superficial injuries and open wound, which are also seen as frequent upstream diagnoses for both patient groups. All the selected downstream diagnoses have an *OR* > 2 and MCC values that vary between 0.11 and 0.43, indicating a positive correlation with ALC. The diagnoses most strongly correlated with ALC are ‘ascites’ and ‘mental-behavioral disorders due to alcohol’. Compared to the remaining diagnoses, they are the most correlated to the ALC disease. Ascites are an accumulation of fluid in the abdominal cavity, which is most often caused by liver cirrhosis and alcohol abuse, and its development indicates advanced liver disease [46].

**Table 3:** Groups of statistically significant (*p*_*value* < 0.001) downstream diagnoses ranked according to their MCC values. For each diagnosis, we show its values for MCC (Matthews Correlation Coefficient), OR (Odds Ratio), OR_CI (its confidence interval), and COV (Coverage).

ICD10 code	MCC	OR	99.9% OR_CI	COV
**1**. **Diagnoses related to liver complications**				
Ascites	0.43	127.32	85.30-190.05	1,454
Oesophageal varices	0.40	365.31	173.69-768.36	1,214
Hepatic failure, not elsewhere classified	0.34	265.76	123.74-570.81	892
Fibrosis and cirrhosis of liver	0.34	142.08	82.25-245.42	945
Other diseases of digestive system	0.28	24.96	18.96-32.85	814
Other diseases of liver	0.24	66.85	39.12-114.25	518
Chronic viral hepatitis	0.16	17.76	11.76-26.82	290
Malignant neoplasm of liver-intrahepatic bile ducts	0.16	35.96	19.64-65.81	242
Other disorders of circulatory system in diseases classified elsewhere	0.13	696.37	25.61-18929.14	140
Peritonitis	0.12	14.39	8.77-23.61	173
Other disorders of fluid, electrolyte and acid-base balance	0.11	7.15	5.15-9.92	250
**2**. **Diagnoses related to alcohol-overuse**				
Mental-behavioral disorders due to alcohol	0.43	18.33	15.91-21.13	2,223
Other diseases of pancreas	0.17	12.84	9.27-17.78	365
Other and unspecified polyneuropathies	0.11	11.22	7.21-17.46	182
**3**. **Diagnoses related to malnutrition**				
Other anaemias	0.19	7.78	6.29-9.63	618
**4**. **Diagnoses related to injuries**				
Fracture	0.13	2.90	2.53-3.34	993
**5**. **Diagnoses related to upper intestinal mucosal**				
Gastric ulcer	0.15	11.25	7.97-15.88	299
Duodenal ulcer	0.12	10.18	6.9-15.00	221
Gastritis and duodenitis	0.15	9.13	6.81-12.24	360
**6**. **Diagnoses related to sepsis**				
Other sepsis	0.13	5.9	4.54-7.65	355

As for the upstream data, we classify the downstream codes into six main classes: (i) coding of the known complications to liver cirrhosis, but this time after the underlying disease has been discovered; (ii) codes related to alcohol-overuse; (iii) malnutrition; (iv) injuries; (v) upper intestinal mucosal; and lastly (v) sepsis.

In addition to the overlap between the upstream and downstream comorbidities, more diagnoses related to liver complication show up in downstream, together with a new group related to sepsis (Table 3). Four new downstream diagnoses are identified as part of the first group, which are ‘peritonitis’, ‘other disorders of fluid, electrolyte and acid-base balance’, ‘other disorders of circulatory system in diseases classified elsewhere’, and ‘malignant neoplasm of liver-intrahepatic bile ducts’. The latter diagnosis contains ‘hepatocellular carcinoma’, which is a cancer almost exclusively developed as a complication to liver cirrhosis [47, 22]. The diagnosis of other disorders of circulatory system in diseases classified elsewhere contains ‘varices of the oesophagus without bleeding due to disease classified elsewhere’. The diseases classified elsewhere are in this case related to liver cirrhosis. This diagnosis has the highest OR, but weak MCC and Coverage values. The other diagnosis coded under peritonitis contains the condition spontaneous bacterial peritonitis, which is a condition almost exclusively seen as a complication to liver cirrhosis. The last new diagnosis of other disorders of fluid, electrolyte and acid-base balance contains hyponatraemia is a frequent complication to liver cirrhosis from a medical point of view.

Compared to the upstream data, we identify fewer diagnoses in the next four downstream groups (ii–v). All of them are already discovered in upstream. The only diagnosis in the sixth group is ‘other sepsis’, which is only identified in the downstream analysis. Most likely this reflects that cirrhotic patients have an increased tendency to contract infections including the most severe ones.

### Conclusion

In this paper, we presented the—to our knowledge—first study of ALF and ALC using registry data for an entire country aiming to identify up- and down-stream diagnoses. We used a knowledge discovery approach to extract relevant information from patient registry data related to ALD and examined if one can identify patients likely to develop ALF or ALC based on their past medical histories. Stratifying the patients, we found that 499 patients were diagnosed with ALF, which highlights the need for earlier diagnosis of ALD. Through a statistical comparison of ALC patients with matched non-ALD controls, we identified six groups of statistically significant upstream diagnoses, which relate to liver dysfunction, alcohol overuse, malnutrition, trauma and injuries, upper intestinal mucosal, and other diagnoses. We similarly identified downstream diagnoses of ALC, which additionally included other sepsis and more diagnoses related to liver complication. We used four different machine-learning algorithms to test how well future ALD diagnoses can be predicted based on upstream diagnoses. Training models on ALC patients, we achieved good performance on an independent test set (AUC = 0.89). To see if it is possible to identify ALD patients already at the ALF stage, we tested the already trained models on the small number of patients diagnosed only with ALF. While this shows promise, the performance drops considerably compared to ALC (AUC = 0.67 for NaiveBayes); this drop reflects that ALF patients are much less sick and thus harder to identify from upstream diagnoses.

The study was approved by the Danish Data Protection Agency [SUND-DT-2017-57] and the Danish Health Authority [FSEID-00003092].

## Supporting information

S1 File The Supporting information file. This file includes supplementary figures, tables and equations of the main manuscript.
(PDF)

S2 File Artificial registry data about individual patients. This table gives an overview about the input data for analysis. We created artificial data for five fictive patients, to give the reader as clear a view of the nature of the data as we can, given the legal constraints patient-sensitive data.
(XLSX)
