# Supplementary material for: Alcoholic liver disease: A registry view on comorbidities and disease prediction
Source: PLoS Comput Biol. 2020 Sep 22;16(9):e1008244. doi: 10.1371/journal.pcbi.1008244 (PMC7531835; doi:10.1371/journal.pcbi.1008244)
Supplement: S1 File — This file includes supplementary figures, tables and equations of the main manuscript. (PDF) [file pcbi.1008244.s001.pdf]

S1 File: Supporting information for the paper "Alcoholic liver disease: A registry view on comorbidities and disease prediction"

Dhouha Grissa, Ditlev Nytoft Rasmussen, Aleksander Krag, Søren Brunak, Lars Juhl Jensen

September 10, 2020

1 Equations

The Odds Ratio (OR) is given by,

$$OR = \frac{Odds\ that\ a\ case\ was\ exposed}{Odds\ that\ a\ control\ was\ exposed} = \frac{a/c}{b/d} \tag{1}$$

The Coverage (COV) is given by,

$$COV = Number\ of\ patients\ with\ the\ key\ diagnosis = a \tag{2}$$

The Matthews Correlation Coefficient (MCC) is given by,

$$MCC = \frac{(a \times d - b \times c)}{\sqrt{(a+b) \times (a+c) \times (d+b) \times (d+c)}} \tag{3}$$

2 Supplementary Figures and Tables

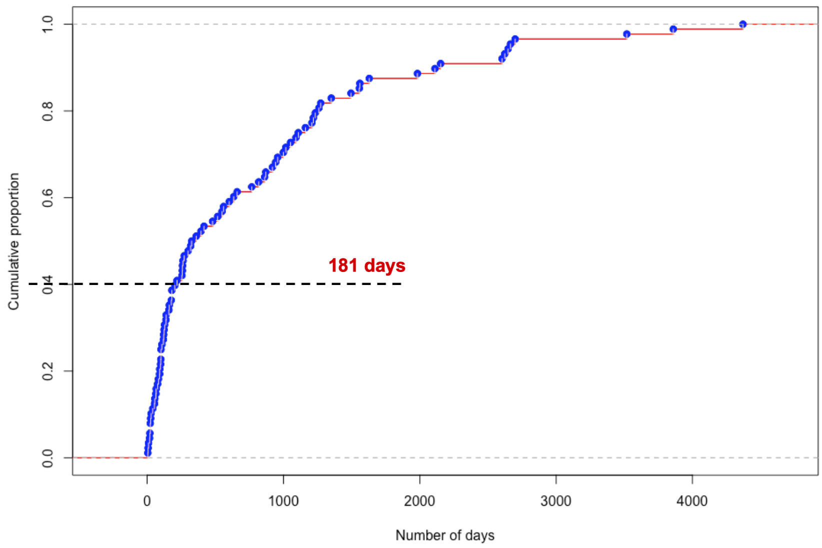

Figure A. ***The cumulative distribution of the 88 patients with ALF and ALC.***

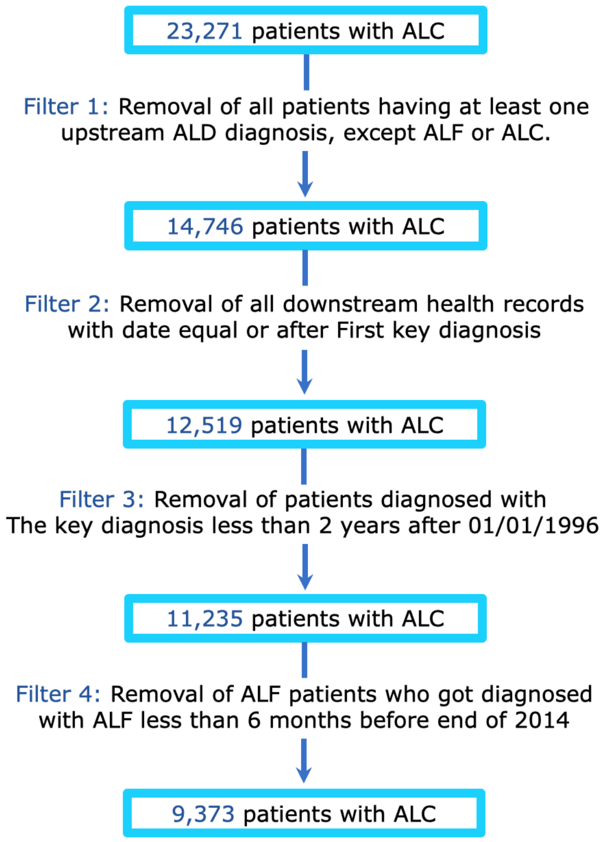

Figure B. *The Flowchart of number of patients with ALC when applying the different filtering criteria.*

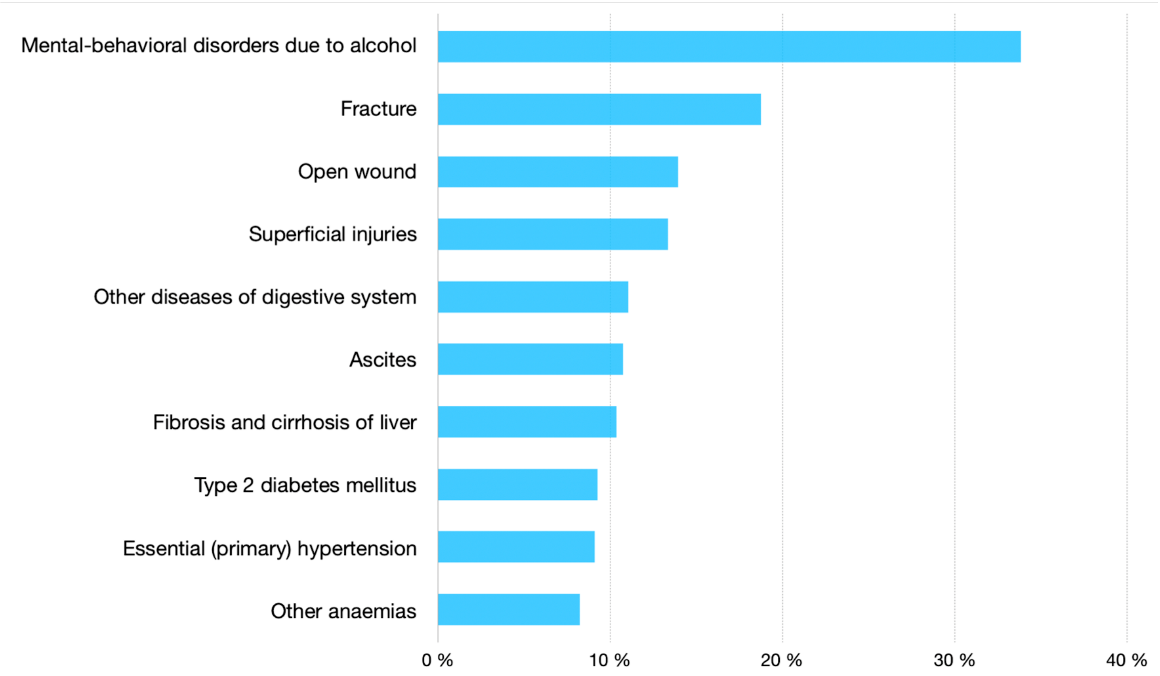

Figure C. *The histogram of most frequent upstream ICD-10 codes among patients with ALC.*

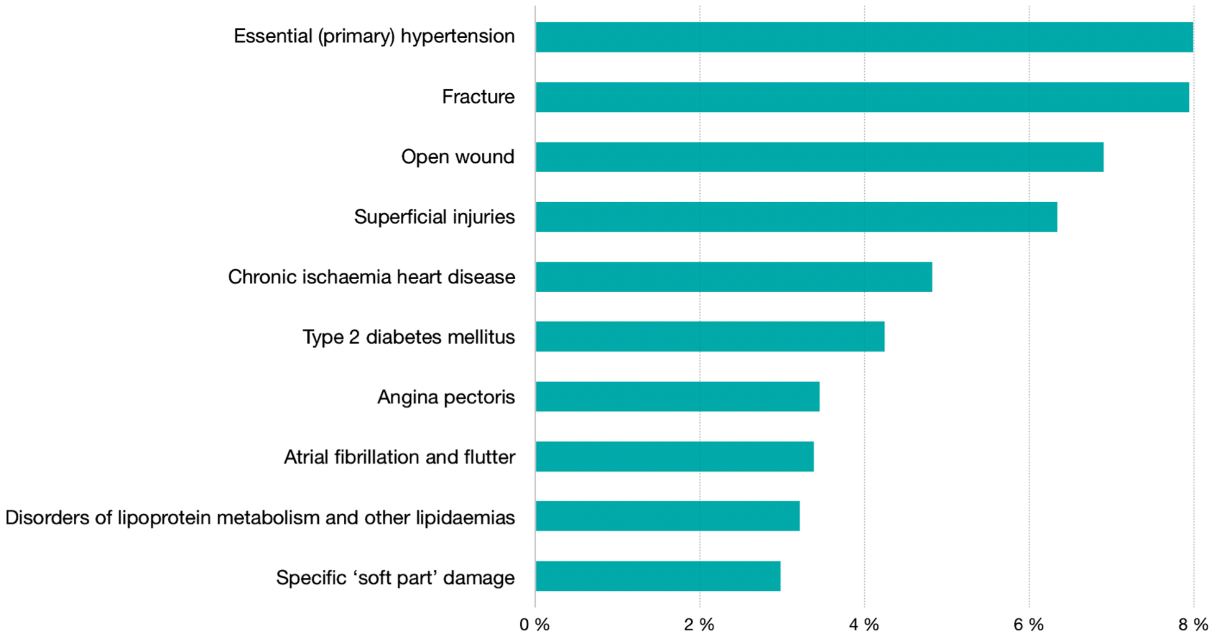

Figure D. ***The histogram of most frequent upstream ICD-10 codes among patients with non-ALD.***

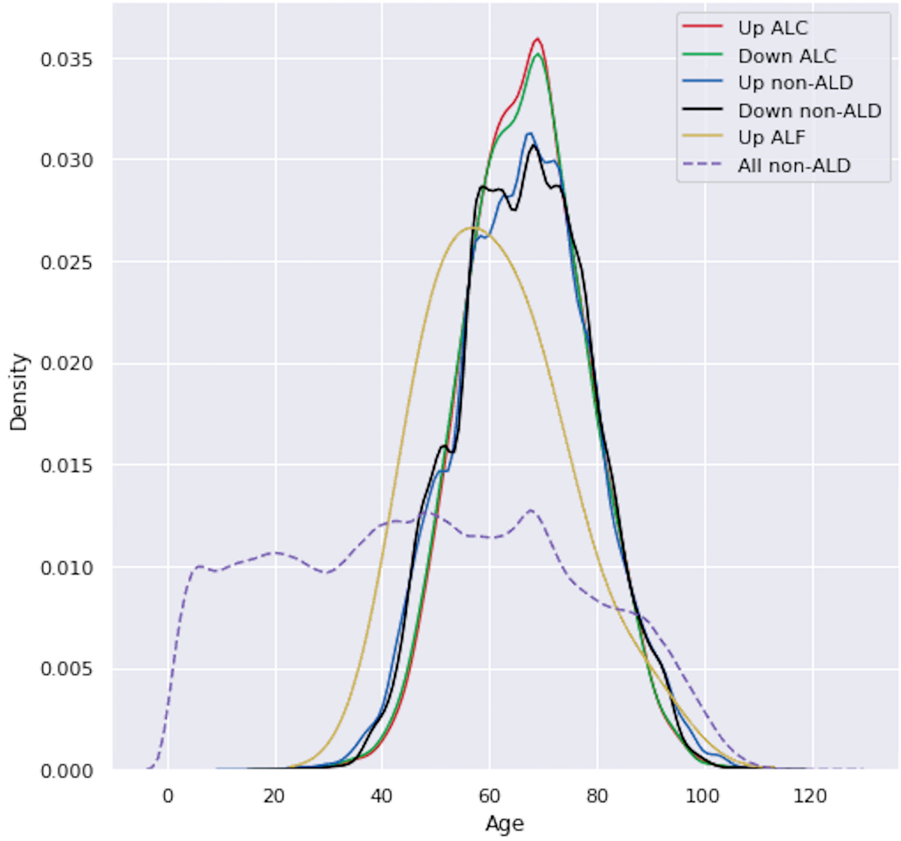

Figure E. ***The distribution of the age ratio among all the discovered groups of patients (ALC, All non-ALD, non-ALD and ALF) both upstream and downstream.***

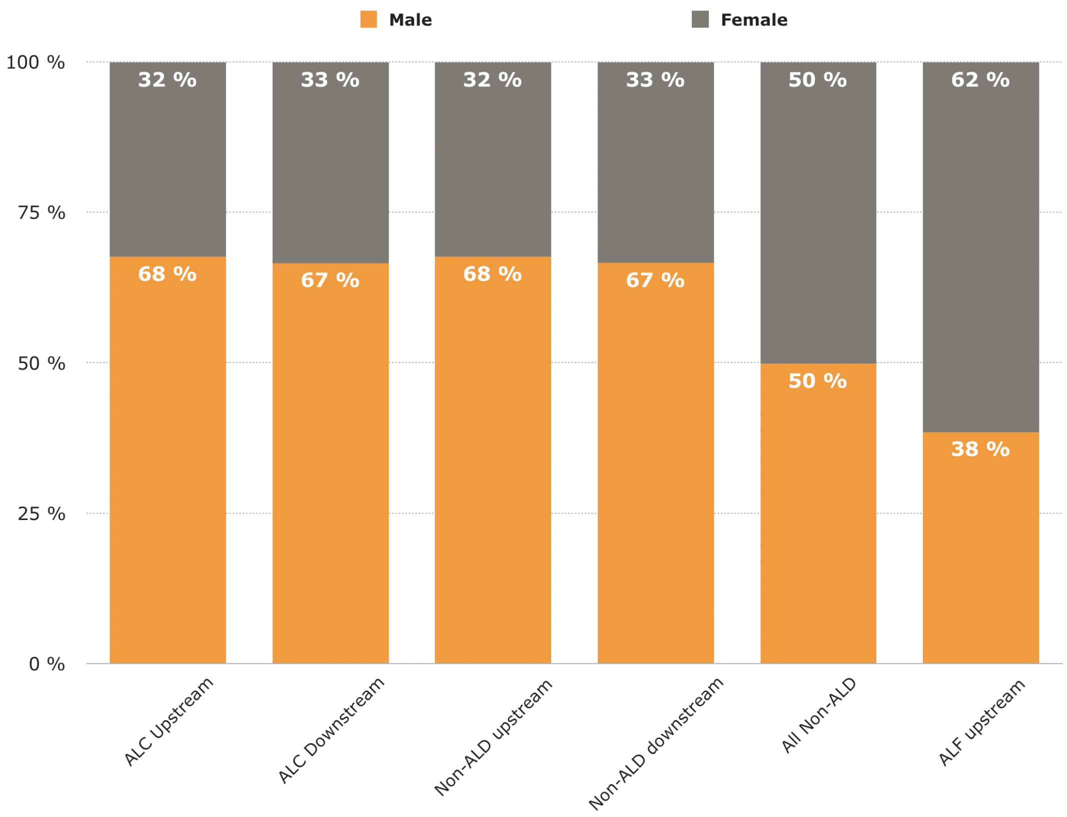

Figure F. *The distribution of the sex ratio among all the discovered groups of patients (ALC, All non-ALD, non-ALD and ALF) both upstream and downstream.*

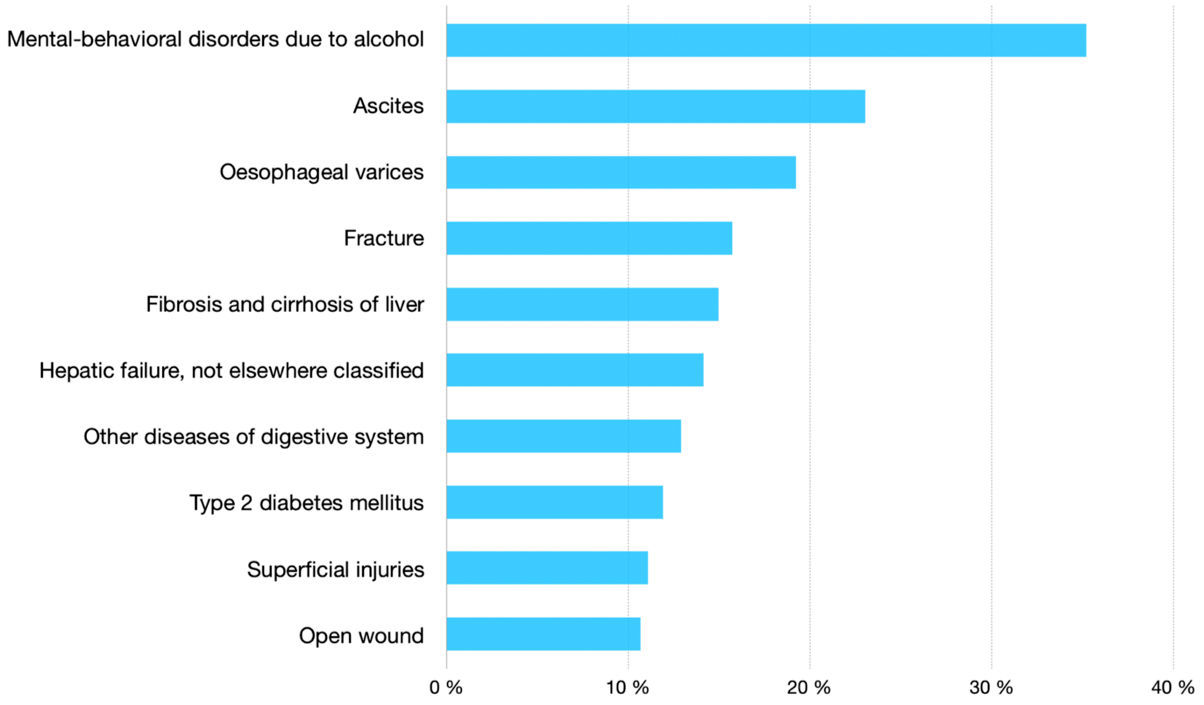

Figure G. *The histogram of most frequent downstream ICD-10 codes among patients with ALC.*

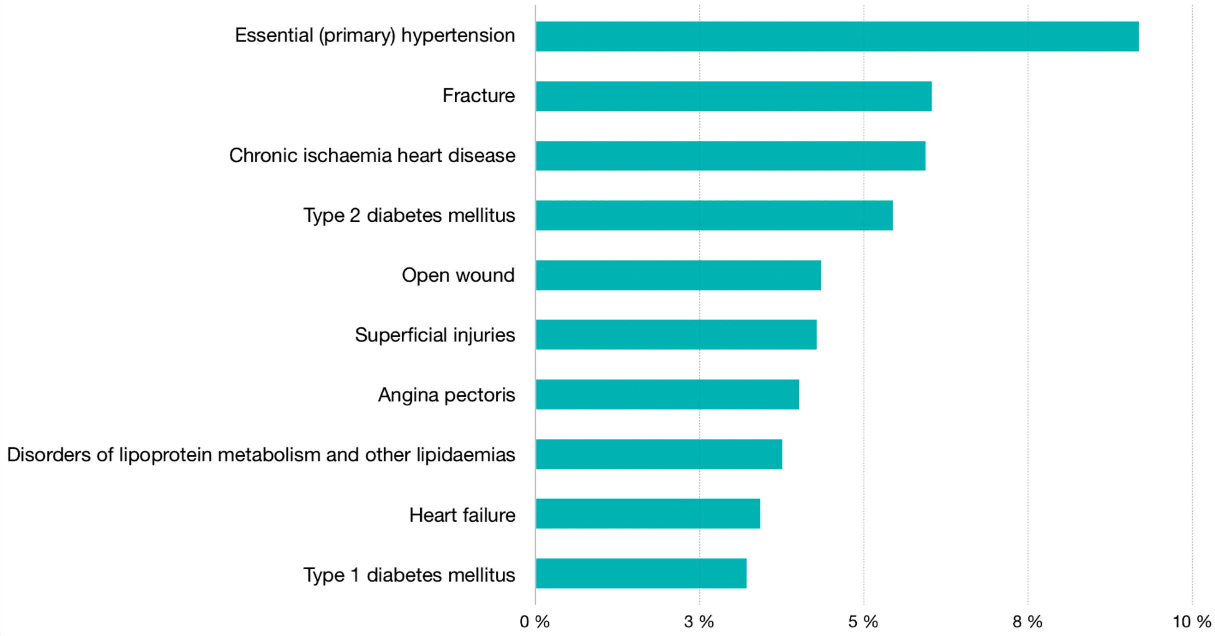

Figure H. ***The histogram of most frequent downstream ICD-10 codes among patients with non-ALD.***

Variable description	Variable name	Variable type	Explanation
Personal identifier	**UNIQ_ID**	string	A unique ID that is given to each patient
Admission identifier	**ADMISSION_ID**	numeric	An ID that is unique to each entry the patients has to the hospital
Records number	RECORD_NUMBER	numeric	Also unique for each entry the patients has to the hospital
Type of hospital encounter	**PATIENT_TYPE**	qualitative	Describes whether the patient is an in-hospital (0/1), out-hospital (2) or emergency-department (3) patient
Admission date	**ADM_DATE**	date	The date of admission
Discharge date	**DIS_DATE**	date	The date of discharge
Hospital owner	**OWNER**	categoric	Whether the hospital is public or private
Hospital identifier	HOSPITAL_ID	numeric	ID of the hospital the patient is treated at
Ward identifier	WARD_ID	numeric	The ward of the hospital the patient is treated at
ICD10 code	**CODE**	string	The ICD-10 diagnosis code (in the Danish system, theres a 'D' placed in front of the ICD-10 code)
Code type	**CODE_TYPE**	string	Whether it is an A 'primary', B 'secondary', H 'referral' or any other code
Source table	**SOURCE_TABLE**	categoric	describes whether the entry is about a diagnose, a screening etc
Number of contact days	CONTACT_DAYS	numeric	The number of days the patient have been at the hospital for this specific entry

Table A. ***The main 13 variables in the Danish National Patient Register.*** *The key variables are the Civil Personal Register (CPR) number (a unique personal identifier used in all Danish registries), the discharge date (Dis_date), and the diagnosis code (a unique code designing each diagnosis or disease). This code is developed by the International Classification of Diseases (ICD), version 10th. Only variables with names in bold were used in this manuscript.*

Chapters removed from the study		
**ICD10 code**	**Chapter description**	**Chapter**
DH00 - DH59	Diseases of the eye and adnexa	VII
DJ00 - DJ99	Diseases of the respiratory system	X
DL00 - DL99	Diseases of the skin and subcutaneous tissue	XII
DO00 - DO99	Pregnancy, childbirth and the puerperium	XV
DP00 - DP96	Certain conditions originating in the perinatal period	XVI
DQ00 - DQ99	Congenital malformations, deformations and chromosomal abnormalities	XVII
DR00 - DR99	Symptoms, signs and abnormal clinical and laboratory findings, not elsewhere classified	XVIII
DV01 - DY98	External causes of morbidity and mortality	XX
DZ00 - DZ99	Factors influencing health status and contact with health services	XXI
**ICD-10 codes removed from the study**		
**ICD10 code**	**Level 3 codes**	**Chapter**
DS07	Crushing injury of head	XIX
DS17	Crushing injury of neck	XIX
DS27	Injury of other and unspecified intrathoracic organs	XIX
DS37	Injury of urinary and pelvic organs	XIX
DS47	Crushing injury of shoulder and upper arm	XIX
DS57	Crushing injury of forearm	XIX
DS67	Crushing injury of wrist and hand	XIX
DS77	Crushing injury of hip and thigh	XIX
DS87	Crushing injury of lower leg	XIX
DS97	Crushing injury of ankle and foot	XIX
DS08	Traumatic amputation of part of head	XIX
DS18	Traumatic amputation at neck level	XIX
DS28	Crushing injury of thorax and traumatic amputation of part of thorax	XIX
DS38	Crushing injury and traumatic amputation of part of abdomen, lower back and pelvis	XIX
DS48	Traumatic amputation of shoulder and upper arm	XIX
DS58	Traumatic amputation of forearm	XIX
DS68	Traumatic amputation of wrist and hand	XIX
DS78	Traumatic amputation of hip and thigh	XIX
DS88	Traumatic amputation of lower leg	XIX
DS98	Traumatic amputation of ankle and foo	XIX
DS09	Other and unspecified injuries of head	XIX
DS19	Other and unspecified injuries of neck	XIX
DS29	Other and unspecified injuries of thorax	XIX
DS39	Other and unspecified injuries of abdomen, lower back and pelvis	XIX
DS49	Other and unspecified injuries of shoulder and upper arm	XIX
DS59	Other and unspecified injuries of forearm	XIX
DS69	Other and unspecified injuries of wrist and hand	XIX
DS79	Other and unspecified injuries of hip and thigh	XIX
DS89	Other and unspecified injuries of lower leg	XIX
DS99	Other and unspecified injuries of ankle and foot	XIX

Table B. ***The list of chapters and codes that were judged as irrelevant by medical doctors and thus removed from the registry data before analysis.***

Level 4 ICD-10 codes			
**ICD10 code**	**Code description**	**Block**	**Chapter**
DK700	Alcoholic fatty liver	Diseases of liver (K70-K77)	XI
DK701	Alcoholic hepatitis	Diseases of liver (K70-K77)	XI
DK702	Alcoholic fibrosis and sclerosis of liver	Diseases of liver (K70-K77)	XI
DK703	Alcoholic cirrhosis of liver	Diseases of liver (K70-K77)	XI
DK704	Alcoholic hepatic failure	Diseases of liver (K70-K77)	XI
DK709	Alcoholic liver disease, unspecified	Diseases of liver (K70-K77)	XI
**Merged ICD10 codes**			
**ICD10 code**	**New group**	**Level 3 codes**	**Chapter**
DS00	Superficial injuries	Superficial injury of head	XIX
DS10	Superficial injuries	Superficial injury of neck	XIX
DS20	Superficial injuries	Superficial injury of thorax	XIX
DS30	Superficial injuries	Superficial injury of abdomen, lower back and pelvis	XIX
DS40	Superficial injuries	Superficial injury of shoulder and upper arm	XIX
DS50	Superficial injuries	Superficial injury of forearm	XIX
DS60	Superficial injuries	Superficial injury of wrist and hand	XIX
DS70	Superficial injuries	Superficial injury of hip and thigh	XIX
DS80	Superficial injuries	Superficial injury of lower leg	XIX
DS90	Superficial injuries	Superficial injury of ankle and foot	XIX
DS01	Open wound	Open wound of head	XIX
DS11	Open wound	Open wound of neck	XIX
DS21	Open wound	Open wound of thorax	XIX
DS31	Open wound	Open wound of abdomen, lower back and pelvis	XIX
DS41	Open wound	Open wound of shoulder and upper arm	XIX
DS51	Open wound	Open wound of forearm	XIX
DS61	Open wound	Open wound of wrist and hand	XIX
DS71	Open wound	Open wound of hip and thigh	XIX
DS81	Open wound	Open wound of lower leg	XIX
DS91	Open wound	Open wound of ankle and foot	XIX
DS02	Fracture	Fracture of skull and facial bones	XIX
DS12	Fracture	Fracture of neck	XIX
DS22	Fracture	Fracture of rib(s), sternum and thoracic spine	XIX
DS32	Fracture	Fracture of lumbar spine and pelvis	XIX
DS42	Fracture	Fracture of shoulder and upper arm	XIX
DS52	Fracture	Fracture of forearm	XIX
DS62	Fracture	Fracture at wrist and hand level	XIX
DS72	Fracture	Fracture of femur	XIX
DS82	Fracture	Fracture of lower leg, including ankle	XIX
DS92	Fracture	Fracture of foot, except ankle	XIX
DS04	Specific 'soft part' damage	Injury of cranial nerves	XIX
DS14	Specific 'soft part' damage	Injury of nerves and spinal cord at neck level	XIX
DS24	Specific 'soft part' damage	Injury of nerves and spinal cord at thorax level	XIX
DS34	Specific 'soft part' damage	Injury of nerves and lumbar spinal cord at abdomen, lower back and pelvis level	XIX
DS44	Specific 'soft part' damage	Injury of nerves at shoulder and upper arm level	XIX
DS54	Specific 'soft part' damage	Injury of nerves at forearm level	XIX
DS64	Specific 'soft part' damage	Injury of nerves at wrist and hand level	XIX
DS74	Specific 'soft part' damage	Injury of nerves at hip and thigh level	XIX
DS84	Specific 'soft part' damage	Injury of nerves at lower leg level	XIX
DS94	Specific 'soft part' damage	Injury of nerves at ankle and foot level	XIX
DS05	Specific 'soft part' damage	Injury of eye and orbit	XIX
DS15	Specific 'soft part' damage	Injury of blood vessels at neck level	XIX
DS25	Specific 'soft part' damage	Injury of blood vessels of thorax	XIX
DS35	Specific 'soft part' damage	Injury of blood vessels at abdomen, lower back and pelvis level	XIX
DS45	Specific 'soft part' damage	Injury of blood vessels at shoulder and upper arm level	XIX
DS55	Specific 'soft part' damage	Injury of blood vessels at forearm leve	XIX
DS65	Specific 'soft part' damage	Injury of blood vessels at wrist and hand level	XIX
DS75	Specific 'soft part' damage	Injury of blood vessels at hip and thigh level	XIX
DS85	Specific 'soft part' damage	Injury of blood vessels at lower leg level	XIX
DS95	Specific 'soft part' damage	Injury of blood vessels at ankle and foot level	XIX
DS06	Specific 'soft part' damage	Intracranial injury	XIX
DS16	Specific 'soft part' damage	Injury of muscle and tendon at neck level	XIX
DS26	Specific 'soft part' damage	Injury of heart	XIX
DS36	Specific 'soft part' damage	Injury of intra-abdominal organs	XIX
DS46	Specific 'soft part' damage	Injury of muscle and tendon at shoulder and upper arm level	XIX
DS56	Specific 'soft part' damage	Injury of muscle and tendon at forearm level	XIX
DS66	Specific 'soft part' damage	Injury of muscle and tendon at wrist and hand level	XIX
DS76	Specific 'soft part' damage	Injury of muscle and tendon at hip and thigh level	XIX
DS86	Specific 'soft part' damage	Injury of muscle and tendon at lower leg level	XIX
DS96	Specific 'soft part' damage	Injury of muscle and tendon at ankle and foot level	XIX

Table C. ***The list of level-4 ICD-10 codes used for ALD and the set of codes that were merged into one unique code.***

Filtering criteria for cases	ALF	ALC
Removal of all patients having at least one of the ALD diseases, except ALF or ALC	x	x
Removal of all downstream health records with assignment date equal or after first date of the key diagnosis	x	x
Removal of all patients who got diagnosed with the key diagnosis less than 2 years after 01/01/1996 (first date of NPR)	x	x
Removal of all patients who got diagnosed with ALF less than 6 months before 31/12/2014 (end of NPR data at hand)	x	-

Table D. ***The list of filtering criteria for the case cohort depending on the outcome of interest*** *(ALF or ALC). In total, four filtering steps were applied in order to extract the most suitable case–control cohorts required for the ALD study analysis.*

		Diagnosed with Target Disease?	
		**Yes**	**No**
Diagnosis X?	Yes	a	b
	No	c	d

Table E. ***A*** 2×2 ***contingency table.*** *The a, b, c and d refer respectively to patients with the disease who got assigned the diagnosis (a), patients without the disease who got assigned the diagnosis (b), patients with the disease who do not got assigned the diagnosis (c) and patients without the disease who do not got assigned the diagnosis (d).*

Years	#patients	#Diagnoses
6 years	8,256	871
5 years	8,777	860
4 years	9,180	856
3 years	9,433	853
2 years	9,373	811
1 year	8,626	750

Table F. ***An overview of the number of patients and diagnoses when changing the size of the time-window from 6 years to 1 year of the upstream data of ALC patients.***

ICD10 code	MCC	OR	COV
Mental-behavioural disorders due to alcohol	**1**	**15**	**1**
Fibrosis and cirrhosis of liver	**2**	**2**	**7**
Ascites	**3**	**5**	**6**
Other diseases of digestive system	**4**	**11**	**5**
Oesophageal varices	**5**	**1**	**11**
Other anaemias	**6**	58	**10**
Other diseases of liver	**7**	**6**	**16**
Gastritis and duodenitis	**8**	40	**13**
Hepatic failure, not elsewhere classified	**9**	**4**	21
Gastric ulcer	**10**	47	**15**
Fracture	**11**	171	**2**
Other diseases of pancreas	**12**	45	**18**
Chronic viral hepatitis	**13**	**16**	26
Other and unspecified polyneuropathies	**14**	**17**	28
Duodenal ulcer	**15**	51	22
Oesophagitis	**16**	41	29
Abdominal and pelvic pain	**17**	145	**12**
Symptoms and signs involving appearance and behaviour	**18**	**19**	241
Superficial injuries	**19**	194	**4**
Open wound	**20**	198	**3**
Type 2 diabetes mellitus	26	192	**8**
Other disorders of circulatory system in diseases classified elsewhere	30	**3**	95
Hepatomegaly and splenomegaly not elsewhere classified	35	**7**	96
Chronic hepatitis, not elsewhere classified	38	**9**	102
Toxic liver disease	43	**14**	104
Specific 'soft part' damage	47	200	**14**
Folate deficiency anaemia	53	**8**	134
Other nutritional anaemias	68	**10**	166
Unspecified viral hepatitis	86	**13**	182
Type 1 diabetes mellitus	87	215	**19**
Acute and transient psychotic disorders	100	**20**	192
Nutritional marasmus	112	**12**	212
Heart failure	130	222	**17**
Other abnormalities of plasma proteins	135	**18**	223
Essential (primary) hypertension	224	226	**9**
Chronic ischaemic heart disease	244	227	**20**

Table G. ***The list of most relevant upstream diagnoses, obtained from computing the MCC, OR and COV metrics on the set of ALC patients and their matched controls.*** *This list of* 39 *features is sorted according to the MCC ranking. In each column, we present in bold the features ranked top-20 for every metric.*

Cohort	Patients	Encounters	Codes
	**Upstream**		
Cases (patients with ALC)	9,082	68,029	820
Controls (patients with no-ALD)	43,844	96,728	941
	**Downstream**		
Cases (patients with ALC)	6,311	75,414	794
Controls (patients with no-ALD)	30,696	77,327	909

Table H. ***The number of patients, clinical encounters and unique codes for cases and controls within 2-year upstream and downstream data.***
